# Decolonizing global health—what is the plan?

**DOI:** 10.1093/eurpub/ckad025

**Published:** 2023-06-01

**Authors:** Adelson Guaraci Jantsch

**Affiliations:** Secretaria Executiva da UNA-SUS (SE/UNA-SUS), Universidade Aberta do SUS, Brasília, Brazil

A few months ago, an old friend from my home town sent me a message asking for help. As part of his duties as a faculty in one Brazilian university, he had to take part in a working group aiming to create a new curriculum for the school of medicine. He is a biologist and some concepts were quite new to him, such as evidence-based medicine (EBM)—‘Is this a new trend? Medicine was based on what before? Witchcraft? And what does “patient-first” even mean?!’ I was not familiar with ‘patient-first’, so he told me ‘Having the patient as part of the treatment, I just learnt’. ‘Oh! they are talking about patient-centered care (PCC)’, I realized. Announcing the obvious, he asked me ‘And where was the patient this whole time?’

After laughing for a while, I tried to tell him the history behind these concepts. None of them represented an actual paradigm shift but rather reinforced the importance of practitioners relying both on the best scientific evidence and on having patients’ experiences, values, possibilities and expectations as guiding principles for taking care of their patients. Doctors were not unfamiliar with scientific methods nor they were too arrogant to not listening to their patients’ opinions, before these two concepts appeared. Of course, it was not the case that physicians automatically started to use number needed to treat to prescribe a new medicine nor they realized the importance of shared decisions making. However, by showing how to put EBM and PCC in practice, these concepts have been changing the way medicine is taught and practiced around the world.

Last week, I received an email from my Swedish friend Birger Forsberg asking me to write a comment on a short article he wrote with his colleague Jesper Sundewall about Decolonizing Global Health (DGH). There are many papers calling for DGH but pragmatism is missing in this literature. Forsberg and Sundewall took a different approach, writing four recommendations that deans in schools of public health, decision-makers, funding institutions and researchers can implement to build more equitable partnerships and create a better power balance between high-income (HIC) and low- and middle-income countries (LMIC): (i) Map how global health research funds are distributed in the world; (ii) promote twinned research programs and funds between HIC and LMIC; (iii) universities recruiting new staff also from LMIC; and, finally, (iv) researchers should more actively collaborate with and include colleagues from LMIC in their projects.

EBM^1^ and PCC^2^ made a difference in the universe of medical practice by presenting some pragmatism to the discussion. If that is the case, we could try to reframe PCC and EBM to DGH in the following way:

- ‘Exploring both the disease and the illness experience’ into ‘exploring the disease and what it takes for patients, practitioners and the health care systems to take care of both illness and disease’. And ‘Understanding the whole person’ into ‘understanding the whole context where the project will run’. A global health project on HIV treatment can recruit many patients in Mozambique but what are the plans to translate its results into changes in the way patients with HIV are treated in that country? If the results can improve our understanding about specific aspects of the disease (which can be useful for HIC) without improving the way treatment is delivered for patients with HIV (which is essential for LMIC) then only one side will benefit from this collaboration.
- ‘Finding common ground’ into ‘finding common ground between all collaborators and stakeholders’. This must include not only researchers, but practitioners and patients. Being a researcher is a hard task for everyone and everywhere. Writing articles, analyzing data, getting updated, learning new research techniques, submitting for grants, networking and teaching are all part of the same job. For those working in LMIC, this job is usually done after hours, and sometimes after a week full of patients.
- ‘Incorporating prevention and health promotion’ into ‘trying not to slice the subject into unrecognizable small pieces—detection, test, treatment (only drugs) or outcomes’. In other words, approach the subject as comprehensible as possible—a mixed methods approach may help.
- ‘Enhancing the patient–doctor relationship’ into ‘enhancing bonds between all actors and institutions involved, making relationships among all parties more horizontal’, including the relationships between researchers and practitioners, managers, decision-makers and politicians.
- ‘Being realistic’ into ’being realistic that patients, families, healthcare providers and healthcare services are the ones that routinely take care of the health condition under investigation’. Again, the disease cannot be detached from its individual, social and healthcare context. Angola can be a promising place for research in end-stage kidney disease. New hemodialysis centers have been built in that country. However, hemodialysis is only one (very important) element in the whole spectrum of renal failure and most of these patients’ health needs are in primary care. With poorly equipped primary care clinics and a model of care organized in vertical programs (1980s GOBI policies), all its risk factors (including malaria, in Angola) will still lack proper management. One study regarding biological aspects of kidney failure will be more helpful for HIC but LMIC will not benefit as much if the main issue in their context (incipient primary care) is missing. This can also be stated using two concepts from EBM as ’Global health projects must shift from a disease-oriented research approach into a patient-oriented^3^ and health systems oriented research approach’.^4^

My old friend from Curitiba summarized EBM and PCC saying that ‘Medicine discovered science. Medicine discovered the patient’. Funny but untrue. They are all parts of a historical process towards better healthcare, not the invention of a new healthcare. What is true though is that both Medicine and Global Health are practiced by human beings who need guidance, boundaries and rules to constrain their actions. Pragmatically, a guideline for reporting global health projects—like CONSORT for trials—could turn these recommendations into standards of good practice. That may set the bar for future projects, grants and publications in global health.

*Conflicts of interest*: None declared.

## References

[1] Evidence-Based Medicine Working Group. Evidence-based medicine. A new approach to teaching the practice of medicine. JAMA 1992;268:2420–2425. 10.1001/jama.1992.03490170092032. 1404801

[2] Stewart M , Brown JB, Weston W, et al Patient-Centered Medicine: Transforming the Clinical Method. 3rd Ed. Boca Raton: Radcliffe Medical Press, 2014; 376 p.

[3] Sacristán JA. Patient-centered medicine and patient-oriented research: improving health outcomes for individual patients. BMC Med Inform Decis Mak 2013;13:6. 23294526 10.1186/1472-6947-13-6 PMC3575265

[4] World Health Organization. What is Health Policy and Systems Research (HPSR)? [Internet]. The Alliance for Health Policy and Systems Research [date accessed: 16 December 2022]. Available at: https://ahpsr.who.int/what-we-do/what-is-health-policy-and-systems-research-(hpsr)

